# The American Dental Association

**Published:** 1887-05

**Authors:** 


### THE AMERICAN DENTAL ASSOCIATION.

A desire seems to be manifest upon the part of many of its most earnest members that there should be no meeting of the American Dental Association this year. We cannot but think that this would be a great mistake, and hope that the subject will be well considered before any decisive action is taken by the Executive Committee. We fully realize the conditions under which the meeting would be held, but cannot avoid the conviction that anything is better than to let a meeting go by default, and the following are some of the reasons that should be carefully weighed.

When an annual meeting is passed over entirely for any reason, it makes a hiatus in the record. What old member does not regret that, even in the youth of the society—in 1861—although the country was then just plunging into the horrors of the War of Secession, a few faithful ones did not meet and hold a meeting, and thus preserve unbroken the list of the annual sessions. And how infinitely less than in 1861 is the excuse for now passing a meeting.

When a meeting fails utterly, and when no effort is made to hold the regular sessions, it argues a lukewarm feeling on the part of the members, and it is very discouraging and disheartening to many of those who have the best interests of the Association at heart.

If no meeting be held, it affords an opportunity for reproach on the part of those who have no desire to see the Association successful. Other societies step into the field and get the attention of the dentists, and draw them away from their allegiance to the old society.

The hour of trial and difficulty is the time when those who are true to the old society should put forth their utmost efforts. In view of the distracting influence of the coming Congress, the Southern Association is making more than usual exertions to obtain a good meeting, and therein the members show the honest character of their allegiance.

Some members of the American Dental Association will not attend the Congress, and are not among its supporters. By what right are they deprived of the meeting to which they annually look forward, and who can blame them, when they see the old organization sacrificed to the international meeting, if they quite withdraw from the former?

An interregnum of a year in the meetings of the American Dental Association means the weakening of its influence upon dentists generally, for it will be an admission that it has not sufficient vitality to hold its own against a meeting which has never been held in this country before, and perhaps may never come here again. Our society should have sufficient vigor to stand in the face of any meeting whatever, and if it be not, it should go to the wall entirely, and be succeeded by an association that is stronger in the confidence and affection of its members.

The Medical Congress meets with us but for this year, while the American Dental Association must be depended upon to maintain the status of dentistry continuously. In this respect it is the more important meeting, and should not be sacrificed to anything whatever. If our dentists cannot decently support both, they should have taken no part in the Congress. Those who are connected with that meeting have no right to offer up our national organization upon its altar.

The selection of Asheville was an unfortunate one for this year, but it was made in the hope that the Southern Dental Society would unite with the American Dental Association in the holding of one meeting for both societies. This the Southern Society declines, and from the society standpoint they are undoubtedly right. Shall the members of the American Dental Association exhibit less loyalty?

Nothing will be lost by holding the meeting, while much may be gained. If members can go to but one, let them attend the Congress. Those who stay away from the Association will be no worse off than if no meeting were to be held, while those who can conveniently attend both will be so much the gainers.

We are decidedly of the opinion that a meeting should be held, even though it were known in advance that not a dozen members would be present. But the meeting should be at some central point, easy of access, and where there can be no charge of favoriteism in the selection. Niagara has always been the compromise point, and we do not think that a better selection could be made this year; but Cleveland, Detroit, and perhaps other places, would be quite unobjectionable.

It should be primarily understood that the change in locality was solely because of the peculiar circumstances of the case, and the

Association should consider itself in honor bound to go to Asheville next year. It is not best to go south when the chances of a large meeting are in any way doubtful, because it would arouse invidious comparisons. So if the place were changed this year, it should be made a point of honor that the Association go to Asheville next year.

The largest meetings are not always the best ones, and if one be held this year it will be a preparation for the Congress, and will enhance the value of that occasion. Let us not allow the good old Association to be shoved to the wall and its meetings to go by default, but let those who will, meet at some central place, prove their loyalty to our national organization and preserve the succession of its anniversaries.

